# A narrative review of approved and emerging anti-obesity medications

**DOI:** 10.1016/j.jsps.2023.101757

**Published:** 2023-08-24

**Authors:** Semira Abdi Beshir, Asim Ahmed Elnour, Aadith Soorya, Affana Parveen Mohamed, Sheron Sir Loon Goh, Nadia Hussain, Amal H.I. Al Haddad, Faizah Hussain, Israa Yousif Khidir, Zainab Abdelnassir

**Affiliations:** a Department of clinical pharmacy and Pharmacotherapeutics, Dubai Pharmacy College For Girls, Dubai, United Arab Emirates; b Program of Clinical Pharmacy, College of Pharmacy, Al Ain University, Abu Dhabi Campus, United Arab Emirates; c AAU Health and Biomedical Research Centre, Al Ain University, Abu Dhabi, United Arab Emirates; d College of Medicine, Gulf Medical University, Ajman, United Arab Emirates; e College of Pharmacy, Gulf Medical University, Ajman, United Arab Emirates; f Department of Primary Care Medicine, Faculty of Medicine, University of Malaya, Kuala Lumpur, Malaysia; g Department of Pharmaceutical Sciences, College of Pharmacy, 105949, Al Ain University, Al Ain, United Arab Emirates; h AAU Health and Biomedical Research Center, Al Ain University, Abu Dhabi, United Arab Emirates; i Chief Operation Officer, Sheikh Shakhbout Medical City (SSMC), Abu Dhabi, United Arab Emirates; j Department of clinical pharmacy and Therapeutics, Dubai Pharmacy College, Dubai, United Arab Emirates; k Department of Clinical Pharmacy and Pharmacy Practice, (PhD, MSc, B Pharm), College of Pharmacy, University of Hail (UOH), Saudi Arabia; l Fourth-year pharmacy, Abu Dhabi, United Arab Emirates; m College of Pharmacy, Al Ain University, Abu Dhabi Campus, United Arab Emirates

**Keywords:** Anti-obesity medications (AOM), Liraglutide (Saxenda), Naltrexone-bupropion (Contrave), Obesity, Orlistat (Xenical), Overweight, Phentermine (Ponderax), Phentermine-topiramate (Qsymia), Semaglutide (Wegovy), Setmelanotide (IMCIVREE), Tirzepatide (Mounjaro), Type 2 diabetes mellitus (T2DM)

## Abstract

**Background:**

Recently, many drugs have been approved for halting overweight and obesity—few types of research shifted to using Anti-obesity medications (AOM) solely for well-being and shape-keeping.

**Objective:**

This narrative review's objective was to explore the use of AOM in relation to their medical indications, efficacy, and cardiovascular safety.

**Methods and materials:**

We have conducted a narrative review of the literature on approved/non-approved AOM used for obesity and overweight. We have shed light on the emerging trials of therapies and evolving remedies.

**Results:**

Recently, there has been an enormous change in the use of AOM with high consumption that deserves extensive surveillance for the long-term consequences and impact on social, mental, and physical health. Nearly six AOMs and combined therapy are approved by the Food and Drug Administration. The recent guidelines for obesity management have shifted the focus from weight loss to goals that the patient considers essential and toward targeting the root cause of obesity.

**Conclusion:**

The use of AOM increased enormously despite its sometimes-dubious safety and ineffectiveness. The public and medical professionals should be vigilant to the real-world benefits of anti-obesity drugs and their achieved effectiveness with an improved safety profile.

## 1 Background

Obesity is the fifth leading cause of death worldwide. Obesity is a lifestyle disease associated with the lack of an individual’s ability to adopt healthy habits (Safaei et al., 2021). However, multiple factors, such as genetic, environmental, behavioral (excessive calorie intake, stress, insomnia), and sociocultural factors, can affect the development of excess fat in the body (Konttinen, 2020, Tappia and Defries, 2020). Although some risk factors are modifiable, others are difficult to control. Some examples of modifiable risk factors for obesity include lifestyle and behavioral factors such as poor dietary habits, sleep deprivation, sedentary lifestyle, and excessive weight and socioeconomic factors. Non-modifiable risk factors for obesity, on the other hand, include genetics, macrosomia at birth, gender, ethnicity, and age (Zawaly et al., 2022).

Excessive weight gain represents dysregulation of complex interactions between the brain and other organs, such as the gastrointestinal system, muscles, and fat cells (adipose tissue) (Wen et al., 2022). The excitatory or suppressant part involves appetite and energy expenditure involving hormones, neurotransmitters, and genes that are implicated in the development of obesity. Genetic mutation of the FTO gene, Melanocortin 4 receptor (MC4R) genes, or P38 MAPK pathway, have been linked with excessive body fat. Several hormones are involved in the regulation of feeding and energy. For instance, adiponectin and leptin hormones produced by adipose tissue activate a melatonin-stimulating hormone that decreases appetite and energy intake (Obradovic et al., 2021). Hence, low adiponectin levels are linked with insulin resistance and obesity (Zhao et al., 2021, Wen et al., 2022). Similarly, Peptide YY, pancreatic polypeptide, glucagon-like peptide 1, oxyntomodulin, and cholecystokinin send inhibitory signals for calorie intake. In contrast, Ghrelin, referred to as a hunger hormone that increases food intake, and the neuropeptide Y (NPY) increases the risk of developing obesity. In addition, adipose tissue releases tumor necrosis factor α and interleukin 6, which are pro-inflammatory factors linked with increased risk of obesity, CVD, and insulin resistance. Excessive fat intake has been associated with dysfunction of gut microbiota, insulin resistance, and hypothalamic circadian rhythm disruptions (Seong et al., 2019), increasing the worsening of obesity. In addition, stress and disturbances in rhythm disruptions also increase the risk of obesity. Stress hormones such as glucocorticoids can increase appetite and food consumption. Other factors, such as adenovirus infection, maternal smoking (Yamashita et al., 2023), and macrosomia at birth, have been linked with obesity. A sedentary lifestyle, lack of physical activity, and unhealthy eating habits are major contributors to obesity. Overconsumption of high-calorie, high-fat, and high-sugar foods, as well as sugary drinks, can lead to weight gain (Wright and Aronne, 2012).

Being overweight or obese increases the risk of medical, economic, and social problems. Excessive body weight is associated with cardiovascular disease (CVDs), stroke, type 2 diabetes mellitus (T2DM), hypertension, chronic kidney disease (CKD), gastroesophageal reflux disease (GERD), polycystic ovarian syndrome (PCOS), infertility, obstructive sleep apnea (OSA), cancer, joint problems, and fatty liver disease. Modest weight loss can reduce the risk of chronic diseases (Jakicic & Jackson, 2021).

**Fig. 1:**
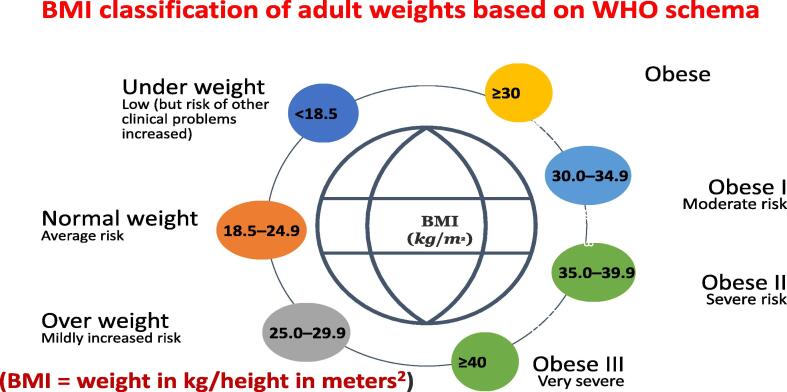
The BMI classification of adult weights based on WHO schema.

The World Health Organization (WHO) uses the body mass index (BMI) to define obesity in adults. BMI was used to classify the individuals into different classes based on weight and height. Individuals with a BMI of 25–29.9 are considered overweight, while those with a BMI of 30–39.9 are considered obese (“Obesity and overweight,” 2021). The BMI classification of adult weights based on the WHO schema is shown in [Fig. 1].

Since weight gain occurs gradually, long-term medical weight loss interventions should be administered with lifestyle modifications. While some people can lose and maintain weight without medication, others will require medications and surgical procedures to help them lose and maintain weight (Brown et al., 2019). Diet and exercise are recommended as the initial interventions for overweight patients; they help to lose 3–5% of their weight. The impact of diet alone on weight loss is limited by the counter-regulatory hunger hormone, ghrelin, which brings weight back to the set point. However, to have a significant impact on comorbid medical conditions, a weight loss of 5–15% is recommended (Crowley et al., 2019). Participants must combine lifestyle interventions with pharmacotherapy or bariatric surgery to achieve and maintain weight loss. Bariatric surgery is considered the gold standard intervention in managing obesity since it also reduces the impact of counter-regulatory hormones such as ghrelin. Bariatric surgery helps people lose an average of 25–30% of their body; however, these procedures are underutilized because not all patients are able or willing to undergo bariatric surgery. Hence, medication may be a good alternative (Kawa and Adra, 2023, Kumar et al., 2021).

Currently approved anti-obesity medications (AOM) by the United States of America (USA) Food and Drug Administration (FDA) include liraglutide (Saxenda), naltrexone-bupropion (Contrave), orlistat (Xenical), phentermine-topiramate (Qsymia), semaglutide (Wegovy), setmelanotide (IMCIVREE), and tirzepatide (Mounjaro). When used with lifestyle interventions, these medications result in an average of 5–15% weight loss (Tchang et al., 2000). These medications may act centrally, act peripherally, or both peripherally and centrally (Mechanick et al., 2020). Centrally-acting obesity medications are a type of AOM that affects the central nervous system to reduce appetite and increase satiety. Some examples of centrally-acting obesity medications include Phentermine, Lorcaserin, Bupropion/naltrexone, Semaglutide, and GLP-1 analogs (Mechanick et al., 2020). Some drugs like phentermine/topiramate and naltrexone/bupropion have central and peripheral effects, while orlistat is an AOM that solely acts peripherally. Regardless of the need to treat overweight, obese people, many healthcare providers remain hesitant to prescribe anti-obesity medications (AOM) due to the concern of poor sustainable efficacy and side effects related to weight control medications. In addition, the high cost, lack of insurance coverage for AOM, and the consideration that excess weight is a behavioral problem can make individuals reluctant to use medications.

### 1.1 Epidemiology causes, and risk factors for overweight and obesity

Even though obesity prevalence is typically higher in older people, schoolchildren, and women, obesity rates continue to rise among both genders and all ages, regardless of location, ethnicity, or socioeconomic level. In some developed countries, the prevalence of obesity has leveled off during the past few years.

Obesity affects more than 30% of the population in the U.S.A. (Luo et al., 2020). The prevalence of overweight and obesity has increased (Chooi et al., 2019). Obesity is relatively uncommon in Asian nations, such as Japan and Korea, with less than 10% of the population being obese; however, it has become increasingly prevalent in the last decade (Fujita et al., 2019). It is predicted that by 2025, 2.7 billion people will be overweight, and nearly, 1 billion people will be obese. Obesity is a global health issue (Townsend et al., 2022).

The authors of the current narrative review noted that children are at a higher risk of being overweight in the United Arab Emirates (UAE). Compared with international standards, obesity is 2.3 times more prevalent in UAE males at age 14 and 1.9 times more prevalent in females, with the p with age (Al-Haddad et al., 2005). Because adult chronic diseases (e.g., CVDs and diabetes) are generally associated with rising obesity rates, the profound public health implications of childhood obesity in children and young adults have dramatically escalated. Loss of 10% weight can lead to significant clinical improvement in several medical conditions (Bakhtiyari et al., 2022, Wright and Aronne, 2012). Understanding the impact of obesity on social, mental, and physical health is crucial for direct clinical management of obesity.

### 1.2 Guideline-based obesity classification and measures of obesity

The American Association of Clinical Endocrinology (AACE) (Garvey et al., 2016), Endocrine Society, Obesity Medicine Association, and Canadian Adult Obesity Clinical Practice Guidelines have provided guidelines for the management of obesity (Pedersen et al., 2022). Recent guidelines for obesity management have shifted the focus from weight loss to goals that the patient considers essential and toward targeting the root cause of obesity. In addition, the focus has shifted to diet- and exercise-based weight-loss programs. New guidelines emphasize the need to treat obesity as a chronic condition owing to either obesity sequelae or the progressive nature of obesity-associated diseases.

According to the AACE guidelines, individuals with a BMI between 25 and 29 and without any weight-related issues may benefit from improving their lifestyle by focusing on an improved diet and increasing physical exercise as their sole therapy strategy. Individuals with a BMI of 27 or above and two or more obesity-related issues are recommended to change their lifestyle and continue to adhere to medications. Medications are recommended if the BMI is ≥ 30, regardless of medical problems. Nutrition and increased physical activity, regardless of BMI, should always be part of an individual’s treatment plan because they enhance cardiovascular conditioning and reduce CVD risk (Wright and Aronne, 2012). Obesity medications can curb your appetite while also increasing your “fullness” signal, which can help subjects adhere to a low-calorie diet. Subjects should consult their medical providers regarding their medical history and potential adverse events of the various AOMs. Surgery was considered for a BMI ≥ 35 with two or more obesity-related comorbidities, in addition to changing one's lifestyle and taking medications. Surgery is considered for patients with a BMI of 40 or higher, regardless of complications.

According to the Endocrine Society guidelines, patients who cannot lose or maintain their desired weight are offered pharmacotherapy. The medication was continued if a patient lost at least 5% of their body weight after three months of therapy. The medication is discontinued, and an alternative medicine or treatment method is sought if the drug is ineffective or if the patient has side effects. People with diabetes who are obese or overweight should be administered medications that encourage weight loss or have no impact on weight as first- and second-line treatments. Several old diabetes medications, such as sulfonylureas, have been linked to weight gain. However, recent remedies such as glucagon-like peptide −1 (GLP-I) analogs and selective glucose transporters-2 (SGLT-2) inhibitors should be considered in all subjects with diabetes who lack contraindications (DiBenedetti et al., 2019). Moreover, phentermine and diethylpropion (appetite suppressants used in the short term) should not be used in subjects with uncontrolled high blood pressure or a history of heart disease (Apovian et al., 2015). The Obesity Medical Association (OMA) provides obesity management algorithms to guide practitioners in treating patients (Obesity Medicine Association, 2013).

### 1.3 The scope, purpose, and rationale of the current narrative review

Many anti-obesity medications have recently received approval for managing overweight and obesity. Phentermine, diethylpropion, benzphetamine, and phendimetrazine are drugs approved for short-term weight management in obese or overweight patients. In contrast, the drugs approved for long-term weight management in subjects with obesity or overweight or with or without a weight-related medical condition include naltrexone-bupropion (Contrave), liraglutide (Saxenda), orlistat (Xenical), phentermine-topiramate (Qsymia), setmelanotide (IMCIVREE), and semaglutide (Wegovy). Other drugs typically used to treat diabetes, such as metformin, zonisamide, and other GLP-1 RAs, are frequently administered “off-label” and at the doctor's discretion to treat obesity. With the recent approval of GLP-1 agonists as more effective anti-obesity medications, the use of w these drugs for medical or nonmedical purposes is increasing worldwide.

### 1.4 Rationale

A considerable proportion of the global population has used these new medications, such as semaglutide and tirzepatide. However, few studies have addressed the shift in the use of drugs solely for well-being and keeping in shape, resulting in a shortage of medicines for those requiring them the most. Therefore, we explored evidence on the effectiveness and safety of approved and other emerging weight-reduction medications. The review gap is based on identifying the long-term consequences and impact of the high consumption of AOMs on social, mental, and physical health.

### 1.5 Objectives of the narrative review

The objective of the current narrative review was to explore the use of AOM for medical indications and other weight-related medical conditions. This review summarizes the evidence on approved AOM and other emerging medications for weight reduction.

## 2 Methods and materials

Google Scholar, Medline, PubMed, Scopus, and Web-of-science were searched to identify relevant publications and ongoing research on AOM. All forms of study designs, including randomized controlled trials (RCTs) and observational trials, were included in the search criteria. We used the main keywords in the search, such as anti-obesity medications, liraglutide (Saxenda), naltrexone-bupropion (Contrave), obesity, orlistat (Xenical), overweight, phentermine (Ponderax), phentermine-topiramate (Qsymia), semaglutide (Wegovy), setmelanotide (IMCIVREE), tirzepatide (Mounjaro); and type2 diabetes mellitus (T2DM). We retrieved the most recent literature on AOM, highlighting the evidence, new emerging molecules, and their safety profiles. The population, intervention, comparator, outcomes, and study design (PICOs) were followed when reporting the included trials [Table 1]. In the current comprehensive narrative review, we summarized and synthesized information from multiple sources, such as research articles, books, guidelines, and other publications. We used a thematic approach and criteria to select and evaluate the included studies. The thematic approach embraces that the requirements for the chosen candidate AOM should include FDA-approved indications for obesity and overweight status. However, we have also shed light on other non-FDA-approved AOMs and the prospects of a new emerging AOM to have the entire scope of obesity and overweight. Second, we explored many trials with a horizon (incomplete or due to completion) to allow more emphasis on the complexity of AOM, extended cardiovascular safety, and the long-term safety of AOM. Lastly, we critically appraised and evaluated the evidence for the efficacy and safety of AOM, highlighting its strengths and weaknesses and offering suggestions for future research on AOM.

**Table 1:** Trials for FDA approved anti-obesity medications (AOM).

**References and trial site**	**Trial start and completion date**	**Study design/ population**	**Intervention/ comparator**	**Percent (%) weight reduction**	**ADR observed and percent (%) of discontinuation**
Jastreboff AM et al. 2022 [**67**]. **SURMOUNT-1**: − 120 study locations - Argentina, Brazil, China, India, Japan, Mexico, Puerto Rico, Russian Federation, Taiwan, USA.	December 2021- April 1, 2022.	RCT, parallel group, double blind study (participant and investigator) **Population**: 2539 People with obesity or BMI 27 kg/m2 and related comorbidities.	**- Intervention**: Tirzepatide: 5, 10, or 15 mg SC, OD a week. **- Placebo**: SC, OD a week.	The mean percentage change in weight at week 72: - - tirzepatide 5 mg weekly = 15.0%. - tirzepatide 10 mg weekly = 19.5%. - tirzepatide 15 mg weekly = 20.9% - Placebo = 3.1%.	- The most common ADR with tirzepatide were gastrointestinal, and the majority were mild to moderate in severity, occurring primarily during dose escalation. - Treatment was discontinued in 4.3%, 7.1%, 6.2%, and 2.6% of participants receiving 5, 10, and 15 mg tirzepatide dosages, respectively, due to adverse effects.
Eli Lilly and Company [**68**]. **SURMOUNT-2** NCT04657003 − 75 study locations - Argentina, Brazil, India, Japan, Puerto Rico, Russian Federation, Taiwan, USA.	Ongoing Start date March 29, 2021 Estimated completion date April 17, 2023.	RCT, parallel-group, double blind study (participant and investigator). **Population**: 900.	**- Intervention**: Tirzepatide: 10 or 15 mg, SC. OD. **- Placebo** SC. OD.	- ongoing trial.	- The estimated study completion is April 17, 2023. - Results still need to be available.
Eli Lilly and Company NCT04657016 [69]. SURMOUNT-3: 65 study locations- Argentina, Brazil, Puerto Rico, USA.	Start date March 29, 2021 Estimated completion date May 11, 2023,	RCT, parallel-group, double blind study (participant and investigator). Population: Eight hundred people with obesity or BMI 27 kg/m2 and related comorbidities.	**- Intervention: Tirzepatide SC.** **- Placebo SC.**	- The co-primary endpoints of percent change in body weight and the proportion of people attaining at least a 5% reduction in their baseline bodyweight by week 72.	- Ongoing trial. - Estimated study completion is May 2023 - Results still need to be available.
NCT04660643 [70] SURMOUNT-4 Argentina, Brazil, Puerto Rico, Taiwan, USA.	Start date March 29, 2021, to May 17, 2023	RCT double-blind, placebo- Population: 750 People with obesity or BMI 27 kg/m2 and related comorbidities	**- Intervention: Tirzepatide** **After 36 weeks of treatment with tirzepatide, the SURMOUNT-3 participants will be randomly assigned to continue or switch to a placebo.**	- At week 88, the investigators will assess whether the participants lost, maintained, or regained weight from the point of randomization.	- Ongoing trial. - Results still need to be available.

**References and trial site**	**Trial start and completion date**	**Study design/ population**	**Intervention/ comparator**	**Percent (%) weight reduction**	**ADR observed and percent (%) of discontinuation**
Wilding JP et al., 2021. [**71**]. **STEP 1** 129 sites in 16 countries in Asia, Europe, North and South America.	- June 4, 2018, to March 5, 2021	RCT, interventional, parallel assignment masking: quadruple (participant, care provider, investigator, outcomes assessor). **Population**: 1961 Obese or overweight people with related comorbidities but not diabetes.	**- Intervention**: Semaglutide - OD weekly SC and diet and physical activity counseling for 68 weeks. - With dose escalation of semaglutide. **- Placebo**: SC.	- Semaglutide: average 14.9% reduction in body weight from baseline during 68 weeks of treatment with semaglutide 2.4 mg plus a lifestyle intervention. - **Placebo**: a 2.4% reduction in body weight in the placebo plus lifestyle intervention group.	- Gastrointestinal ADR: - disorders (typically nausea, diarrhea, vomiting, and constipation) were the most frequently reported events and occurred in more participants receiving semaglutide than those receiving placebo (74.2% vs. 47.9%). - Serious gastrointestinal disorders (1.4% of participants in the semaglutide group and 0% in the placebo group) and hepatobiliary disorders (1.3% with semaglutide and 0.2% with placebo).- Gallbladder-related disorders (mostly cholelithiasis) were reported in 2.6% and 1.2% of participants in the semaglutide and placebo groups, respectively.
Davies et al., 2021. [**72**]. **STEP 2**One hundred forty-nine outpatient clinics in 12 countries across Europe, North and South America, the Middle East, South Africa, and Asia.	- June 4, 2018, to Nov 14, 2018.	RCT, double-blind, double-dummy, placebo-controlled, phase 3 trial. **Population**: One thousand two hundred ten patients are overweight or obese with type 2 diabetes.	**- Intervention**: semaglutide 2·4 mg (4 0 4).semaglutide 1·0 mg (4 0 1). **- Placebo** (n=403).	- Average bodyweight reductions: - semaglutide 2.4 mg: 9.64%, semaglutide 1.0 mg: 6.99%, and **Placebo**: 3.42%	- the proportion of patients who experienced mild to moderate gastrointestinal ADR on: - - Semaglutide 2·4 mg: 63·5% patients. - Semaglutide 1 mg: 57·5% of patients. -Placebo: 34·3% of patients.
Wadden et al., 2021. [**73**]. STEP 341 sites in the United States	August 2018 to April 2020.	- RCT, double-blind, placebo-controlled, multicentre study for 68 weeks. - Population: Six hundred eleven overweight or obese people with related comorbidities but not diabetes.	**- Intervention: semaglutide 2.4 mg (407 subjects).** **- Placebo (204 subjects).** **Both were combined with a low-calorie diet for the first eight weeks and intensive behavioral therapy (i.e., 30 counseling visits) for 68 weeks.**	- The average weight reduction after 68 weeks from baseline: - Semaglutide 16%. Placebo: 5.7%. The proportion of patients with at least a 5% reduction in body weight. Semaglutide: 86.6% placebo: 47.6%.	- Gastrointestinal ADR: -Semaglutide (82.8%) vs. placebo (63.2%) .
Rubino et al., 2021. [74]. STEP4Seventy-three sites in Denmark (2), Israel (6), Netherlands (3), Portugal (6), South Africa (6), Spain (7), Sweden (4), Switzerland (6), Ukraine (5), and the USA (28).	June 4, 2018, to March 20, 2020.	- RCT, parallel assignment quadruple masking Population: Nine hundred two overweight or obese people with type 2 diabetes have reached the target.	**- Intervention: Semaglutide 2.4 mg Once-weekly in Dose During the run-in Period** **- Placebo** **SC of semaglutide placebo OD-weekly.**	- Participants who continued to take semaglutide after randomization lost an additional 7.9% of their body weight, on average, to give a total 17.4% weight loss over the entire trial. - Patients who switched to placebo regained an average of 6.9%, giving a total weight loss of 5.0%.	- Gastrointestinal ADR: - Semaglutide: 49.1% vs. placebo: 26.1%.

**References and trial site**	**Trial start and completion date**	**Study design/ population**	**Intervention/ comparator**	**Percent (%) weight reduction**	**ADR observed and percent (%) of discontinuation**
Garvey et al., 2022, [**75**] **STEP 5**41 sites in 5 countries as follows: Canada (9 places), Hungary (6 places), Italy (5 locations), Spain (6 locations), and the USA (15 sites).	October 5, 2018, to March 23, 2021.	**RCT, parallel assignment,** Quadruple (Participant, Care Provider, Investigator, Outcomes Assessor) masking trial **STEP 5** tested the durability of weight loss across a full two years of treatment. **Population**: Three hundred four obese or overweight people with related comorbidities but not diabetes.	**- Intervention**: semaglutide 2.4 mg versus once-weekly injection. **- Placebo**: SC of semaglutide placebo OD-weekly.	- Semaglutide resulted in decreasing weight until about week 60, and weight loss was maintained through week 104, at which point there was an average placebo-corrected weight loss of 12.6 percentage points.	- Mild to moderate gastrointestinal ADR: - Semaglutide: 82.2% vs. placebo: 53.9%.
Kadowaki et al., 2022, [**76**]. **STEP 6**Japan and South Korea.	Jan 21, 2019 to November 20, 2020.	RCT, double-blind, double-dummy, placebo-controlled, phase 3a superiority trial. **Population**: 401 obese or overweight East Asian people with related comorbidities	- **Intervention**: Semaglutide 2·4 mg (n=199), semaglutide 1·7 mg (n=101) **- Placebo** (n=101)	- Body weight reductions: - Semaglutide 2.4 mg/week =13.2% Semaglutide 1.7 mg/week= 9.6%, placebo, = 2.1%	- Mild to moderate gastrointestinal ADR: - Semaglutide 2.4 mg: 59% of 199 participants semaglutide 1.7 mg: 64% Placebo: 30%
NCT04251156, [**77**]. **STEP 7** China, Hong Kong, the Republic of Korea, and Brazil.	December 8, 2020, to August 23, 2022.	- RCT, parallel quadruple masking. - **Population**: Three hundred seventy-five overweight or obese people with or without type 2 diabetes.	- **Intervention**: Semaglutide 2.4 mg or placebo for 44 weeks.	- Results not reported	- Completed, results not posted
Rubino et al., 2021. [78]STEP 819 USA sites.	September 2019 to May 2021.	- RCT, open-label, 68-week, phase 3b Population: 338 adults with a BMI of 30 or greater or 27 or greater with one or more weight-related comorbidities, without diabetes	- - Intervention: Semaglutide, 2.4 mg OD-weekly SC (16-week escalation, or: - Placebo, or OD, daily SC liraglutide, 3.0 mg (4-week escalation, or matching placebo, plus diet and physical activity.	- Semaglutide: 15.8% weight loss. - Liraglutide: 6.4% weight loss.	- Gastrointestinal ADR Semaglutide: 84.1%. Liraglutide: 82.7%.

**References and trial site**	**Trial start and completion date**	**Study Design/ Population**	**Intervention/ Comparator**	**Percent (%) weight reduction**	**ADR observed and percent (%) of discontinuation**
Pi-Sunyer X et al., 2015. [**79**] **SCALE trial** USA, Canada, Europe, Australia, and Asia. Specifically, the trial involved 32 countries and 191 study sites.	September 2010 to June 2013.	- **SCALE** obesity and prediabetes is 56 weeks, RCT double-blind. **Population**: 3731 patients who did not have type 2 diabetes and had a BMI of at least 30 or a BMI of 27 if they had treated or untreated dyslipidemia or hypertension.	- **Intervention**: OD, daily SC injections of liraglutide at a dose of 3.0 mg, plus counseling on lifestyle modification. **- Placebo** plus counseling on lifestyle modification.	- % of patients who lost at least 5% of their body weight: - liraglutide group = 63.2% vs. placebo group= 27.1%. - % of patients lost at least 10% of their body weight: -Liraglutide=33.1% vs. placebo: 10.6%.	- The most common ADR of liraglutide was mild or moderate nausea and diarrhea. - Serious ADR occurred in 6.2% of the patients in the liraglutide group and 5.0% in the placebo group.
Garvey et al., 2012. [**80**] **SEQUEL trial**The USA.	December 2008 to June 2010.	RCT, placebo-controlled, double-blind, 52-week extension study **Population**: 866 overweight and obese subjects with ≥2 wt-related comorbidities.	- **Intervention**: Phentermine 7.5/46 mg plus topiramate CR or phentermine 15/92 mg plus topiramate CR. - **Placebo**.	- Least-squares mean percentage changes from baseline body weight were phentermine 15/92 mg plus topiramate CR = −10.5%. - Phentermine 7.5/46 mg plus topiramate CR controlled-release = −9.3%, s. placebo= −1.8%. - Significantly more phentermine 15/92 mg plus topiramate CR-treated subjects at each dose achieved ≥5%, ≥10%, ≥15%, and ≥20% weight loss compared with placebo (P < 0.001).	- Phentermine plus topiramate CR was well tolerated over 108 weeks, with reduced rates of ADR occurring between 56 and 108 weeks compared with rates between 0 and 56 weeks.
Allison DB et al., 2011. [81] EQUIP trial Ninety-three sites across eight countries, including the USA, Canada, United Kingdom, Germany, Netherlands, Sweden, Australia, and New Zealand.	2008 to 2011.	- RCT, double-blind, parallel-group design, placebo-controlled for 56 weeks.Population: 1267 men and women with class II and III obesity (BMI ≥ 35 kg/m2) .	- Intervention: Phentermine plus topiramate CR 3.75/23 mg. Phentermine plus topiramate 15/92 mg CR, added to a reduced-energy diet. Placebo: group, added to a reduced-energy diet.	- Weight loss from baseline body weight at 56 weeks: - Phentermine plus topiramate 3.75/23 mg CR = 5.1%. Phentermine plus topiramate 15/92 mg CR = 10.9%. Placebo =1.6%. - Patients who lose at least 5% body weight phentermine plus topiramate 15/92 mg CR = 66.7% of patients, phentermine plus topiramate 3.75/23 mg CR group = 44.9% of patients. Placebo = 17.3%.	- Most common ADRs were paraesthesia, dry mouth, constipation, dysgeusia, and insomnia.

**References and trial site**	**Trial start and completion date**	**Study Design/ Population**	**Intervention/ Comparator**	**Percent (%) weight reduction**	**ADR observed and percent (%) of discontinuation**
Gadde et al. 2011. [**82**] **CONQUER trial** The USA.	November 2007 to June 2009.	- RCT, placebo-controlled, phase 3. **Population**: 2487 overweight or obese adults with a BMI of 27–45 kg/m2 and two or more comorbidities.	- **Intervention**: - OD-daily phentermine plus topiramate 7.5/46 mg, or OD-daily phentermine plus topiramate 15/92 mg. - **Placebo**	- % of patients achieved at least 5% weight loss: - Phentermine plus topiramate 15/92 mg CR = 70%. Phentermine plus topiramate 7.5/46 mg CR = 62%. **Placebo** =21%. - % patients with ≥10% weight loss: - Phentermine plus topiramate 15/92 mg CR = 48%. Phentermine g plus topiramate 7.5/46 mg CR = 37%. **Placebo** =7%.	- The most common ADRs were dry mouth, paraesthesia, constipation, insomnia, dizziness, and dysgeusia. - phentermine plus topiramate 15/92 mg had depression-related ADR. - The ADR was more common in the phentermine plus topiramate 15/92 mg group than the others.
Greenway FL et al., 2010. [**83**]. **COR-I trial**. Thirty-four sites in the USA.	October 2007 to May 2009.	- a multicentre, randomised, double-blind, placebo-controlled, phase 3 trial **Population:** − 1742 participants aged 18–65 years who had a BMI of 30–45 kg/m2 and uncomplicated obesity or BMI of 27–45 kg/m2 with dyslipidemia or hypertension	- **Intervention**: Naltrexone 32 mg plus bupropion 360 mg, naltrexone 16 mg plus bupropion 360 mg per day **- Placebo**	- Mean change in body weight at 56 weeks naltrexone 32 mg plus bupropion group = −6.1% (SE 0.3) naltrexone 16 mg plus bupropion group = −5.0% (SE 0.3) in the placebo group =1.3% (SE 0.3) - % of patients who decrease in body weight of 5% or more: - naltrexone 32 mg plus bupropion = 48%. naltrexone 16 mg plus bupropion =39%. **placebo** =16%	- Headache, constipation, dizziness, vomiting, and dry mouth were also more frequent in the naltrexone plus bupropion groups than in the placebo group. - A transient increase of around 1.5 mm Hg in mean systolic and diastolic blood pressure was followed by a reduction of about 1 mm Hg below baseline in the naltrexone plus bupropion groups
Torgerson JS et al., 2004. [84]. XENDOS trial Europe, Australia, and New Zealand. The study enrolled patients from 37centers in 11 European countries (Austria, Belgium, Denmark, Finland, Germany, Greece, Ireland, Norway, Spain, Sweden, and the United Kingdom)	1999–2004	- A 4-year, double-masked, prospective study Population: 3,305 participants with MI ≥30 kg/m2	- Intervention: Orlistat 120 mg three times daily plus lifestyle changes. - Placebo, three times daily plus lifestyle changes.	- After four years, mean weight loss: - Orlistat =5.8 kg. Placebo = 3.0 kg. - A second analysis including subjects who dropped out of the study: - Orlistat group = 3.6 kg. Placebo = 1.4 kg.	- The overall incidence of ADR: - Orlistat = placebo group. - During the first year of treatment, the proportion of patients experiencing at least one gastrointestinal event, orlistat =91% vs. placebo = 65%. - During the 4th year: - Orlistat =36 vs. placebo = 23%.- At least one serious adverse event: - Orlistat= 15% vs. Placebo=13%. - Similar proportions of serious gastrointestinal ADR occurred in placebo (n = 32; 2%) and orlistat (n = 32; 2%) groups. - No deaths were attributed to study medication.

**Keys: -ADR**: adverse drug reaction; **BMI**: body mass index; **CONQUER**: Effects of low-dose, controlled-release, phentermine plus topiramate combination on weight and associated comorbidities in overweight and obese adults; **COR-I**: Effect of naltrexone plus bupropion on weight loss in overweight and obese adults; **CR**: controlled-release; **EQUIP**: Controlled-release phentermine/topiramate in severely obese adults: a randomized controlled trial; **i.e.**: that is to say; **MI**: myocardial infraction; **RCT:** randomized controlled trial; **SC**: subcutaneous; **SCALE**: A Randomized, Controlled Trial of 3.0 mg of Liraglutide in Weight Management; **SEQUEL**: Two-year sustained weight loss and metabolic benefits with controlled-release phentermine/topiramate in obese and overweight adults; **STEP1**: Research Study Investigating How Well Semaglutide Works in People Suffering From Overweight or Obesity; **STEP2**: Research Study Investigating How Well Semaglutide Works in People With Type 2 Diabetes Suffering From Overweight or Obesity; **STEP3**: Research Study to Look at How Well Semaglutide is at Lowering Weight When Taken Together With an Intensive Lifestyle Program; **STEP4**: Research Study Investigating How Well Semaglutide Works in People Suffering From Overweight or Obesity; **STEP5**: Two-year Research Study Investigating How Well Semaglutide Works in People Suffering From Overweight or Obesity; **STEP6**: Research Study Investigating How Well Semaglutide Works in People Living With Overweight or Obesity; **STEP7**: Research Study of How Well Semaglutide Works in People Living With Overweight or Obesity; **STEP8**: Research Study to Investigate How Well Semaglutide Works Compared to Liraglutide in People Living With Overweight or Obesity; **SC**: Subcutaneous; **SURMOUNT 1**: A Study of Tirzepatide (LY3298176) in Participants With Obesity or Overweight; **SURMOUNT 2**: A Study of Tirzepatide (LY3298176) in Participants With Type 2 Diabetes Who Have Obesity or Are Overweight; **SURMOUNT 3**: A Study of Tirzepatide (LY3298176) In Participants After A Lifestyle Weight Loss Program; **SURMOUNT 4**: A Study of Tirzepatide (LY3298176) in Participants With Obesity or Overweight for the Maintenance of Weight Loss; **USA**: United States of America; **vs**: versus; **XENDOS**: XENical in the prevention of diabetes in obese subjects.

## 3 Results

**Fig. 2:**
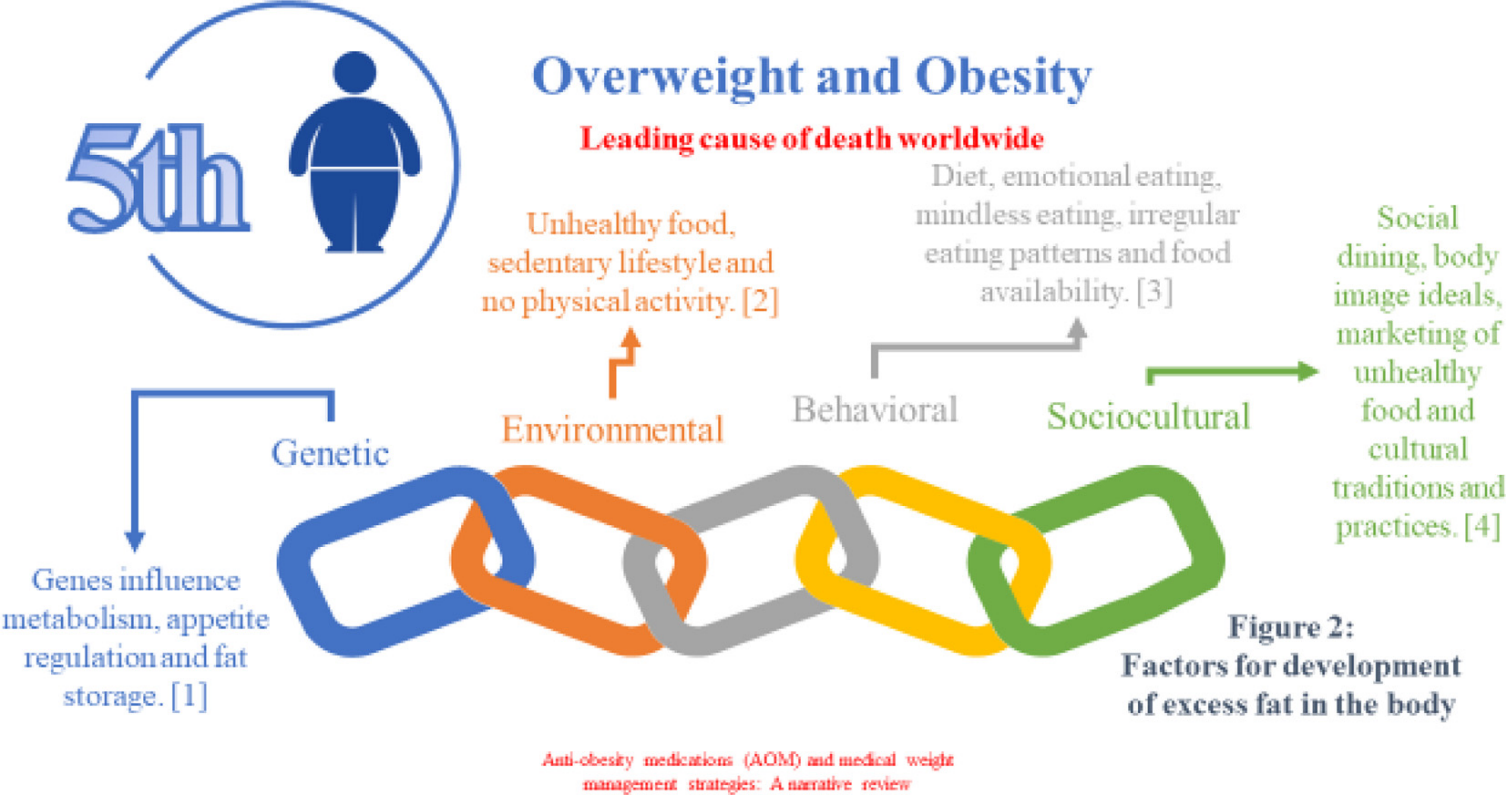
**The f**actors for developing excess fat in the body.

**Fig. 3:**
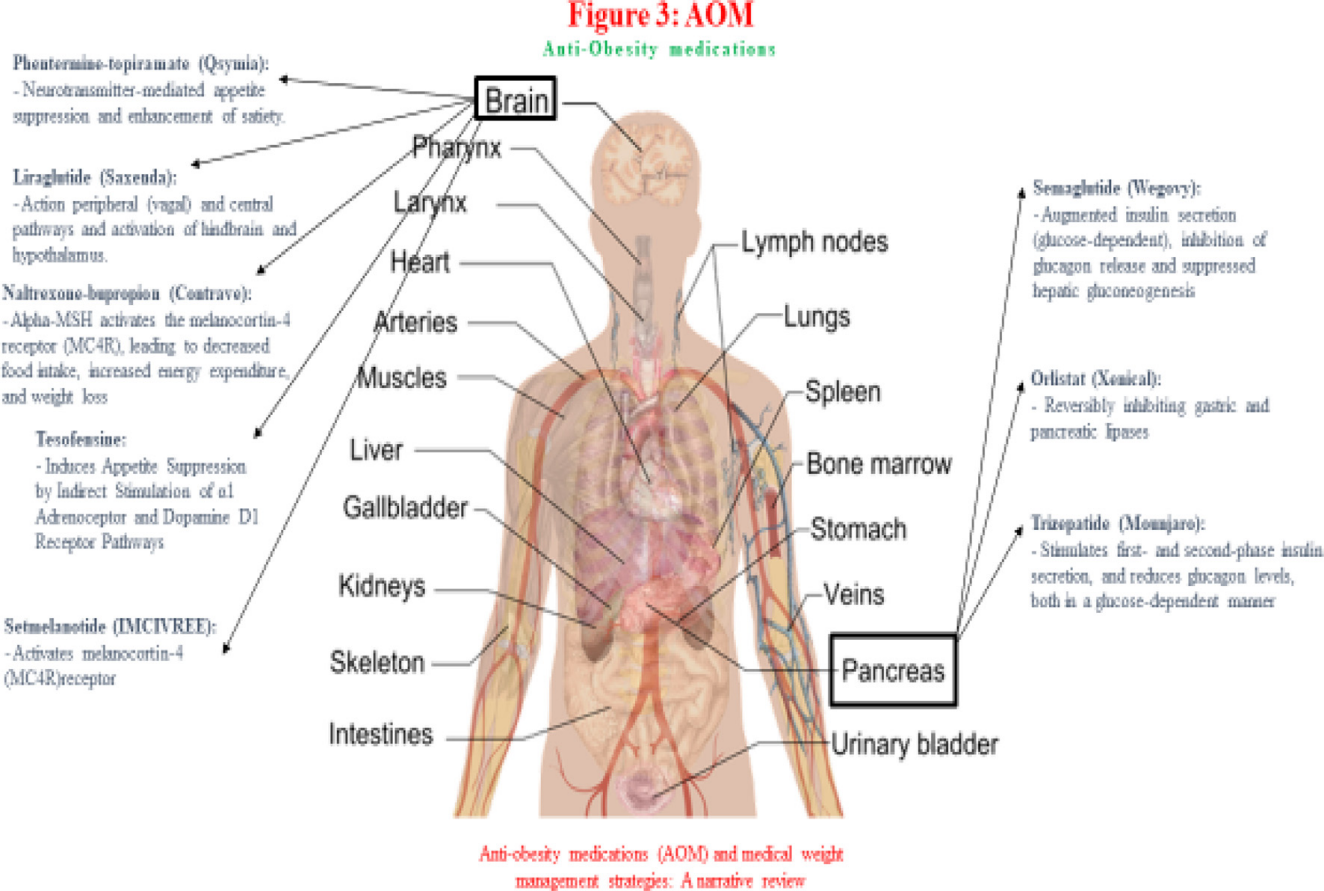
The target of action of AOMs.

**Fig. 4:**
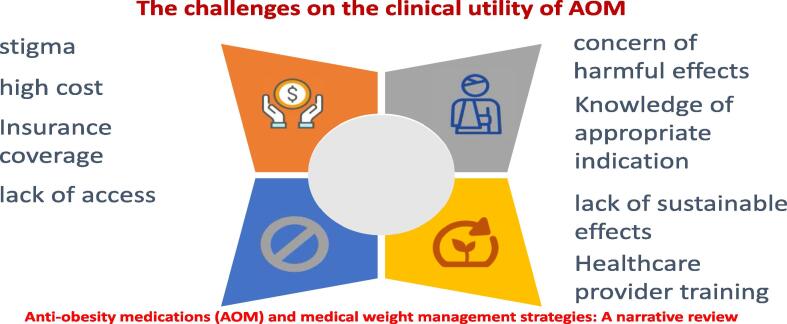
The Challenges on the clinical utility of AOMs.

Obesity is the fifth most common reason for death worldwide. Some known factors for developing excess fat in the body are shown in Fig. 2. Some of the critical AOM trials are presented in [Table 1 and Fig. 3]. However, several challenges hinder the clinical utility of AOM, as shown in [Fig. 4]. Table 2 illustrates the pharmacoeconomic comparative cost analysis of FDA-approved anti-obesity medications (AOM). Table 3 shows the comparison between Ozempic and Wegovy for specific parameters, such as indications and posology. The USA FDA-approved medications for the management of obesity.

**Table 2:** Pharmacoeconomic comparative cost analysis for FDA approved anti-obesity medications (AOM) [**85**].

**AOM non-proprietary and proprietary names**	**US FDA approved AOM**	**Other FDA approved AOM**	**Anti-obesity dose per day**	**Cost ($, EUR)**	**FDA approved indications**
**Lirgaglutide (**Saxenda®)	**USA Approval: 2014**	**–**	Injection 3 mg for use in treating obesity in adolescents (12–17 years) with a body weight above 60 kg and an initial (BMI) corresponding to 30 kg/m2 or greater for adults.	18 mg/ 3 mL Saxenda subcutaneous solution from $1,430.01 for 15 mL.	(Saxenda®) is a (GLP-1) receptor agonist indicated as an adjunct to a reduced-calorie diet and increased physical activity for chronic weight management in adult patients with an initial (BMI) of 30 kg/m2 or greater (obese) (1) or 27 kg/m2 or greater (overweight) in the presence of at least one weight-related comorbid condition (e.g., hypertension, type 2 diabetes mellitus, or dyslipidemia)?
**Semaglutide** (Wagovy®)	**USA Approval: 2017**	–	Injection: a pre-filled, single-dose pen that delivers doses of 0.25 mg, 0.5 mg, 1 mg, 1.7 mg, or 2.4 mg.	Wegovy SC solution (0.25 mg/0.5 mL $1,430 for a supply of 2 mL. **Same price $1,430 for: -** 0.5 mg for 2 mL, 1 mg,/0.5 mL for 2 mL, 1.7 mg/0,75 mL for 3 mL, or 2.4 mg./0.75 mL for 3 mL.	(Wagovy®) is a 1 (GLP-1) receptor agonist indicated as an adjunct to a reduced calorie diet and increased physical activity for chronic weight management in adult patients with an initial (BMI) of 30 kg/m2 or greater (obesity) or 27 kg/m2 or greater (overweight) in the presence of at least one weight-related comorbid condition (e.g., hypertension, type 2 diabetes mellitus, or dyslipidemia)?
**Phentermine** (SUPRENZATM®)	**USA Approval: 1959**	–	They are orally disintegrating tablets containing 15 mg, 30 mg, or 37.5 mg phentermine hydrochloride.	15 mg phentermine oral capsule from $11.68 for seven capsules. 30 mg phentermine oral capsule from $11.25 for seven capsules, 37.5 mg phentermine oral capsule from $22.45 for 15 capsules.	(SUPRENZATM®) is a sympathomimetic amine anorectic indicated as a short-term adjunct (a few weeks) in a regimen of weight reduction based on exercise, behavioral modification, and caloric restriction in the management of exogenous obesity for patients with an initial body mass index greater than or equal to 30 kg/m2, or greater than or equal to 27 kg/m2 in the presence of other risk factors (e.g., controlled hypertension, diabetes, hyperlipidemia)? The limited usefulness of agents of this class, including Suprenza, should be measured against possible risk factors inherent in their use.
**Phentermine-topiramate (**Qsymia®**)**	**USA Approval: 2012**	–	Capsules: (phentermine mg/topiramate mg extended-release) • 3.75 mg/23 mg. • 7.5 mg/46 mg. •11.25 mg/69 mg. • 15 mg/92 mg.	Qsymia oral capsule, extended-release (3.75 mg-23 mg), is around $199 for a supply of 30 capsules.	**(**Qsymia®**)** is a combination of phentermine, a sympathomimetic amine anorectic, and topiramate extended-release, an antiepileptic drug, indicated as an adjunct to a reduced-calorie diet and increased physical activity for chronic weight management in adults with an initial (BMI) of 30 kg/m2 or greater (obese) (1) or 27 kg/m2 or greater (overweight) in the presence of at least one weight-related comorbidity such as hypertension, type 2 diabetes mellitus, or dyslipidemia.

**AOM non-proprietary and proprietary names**	**US FDA approved AOM**	**Other FDA approved AOM**	**Anti-obesity dose per day**	**Cost ($, EUR)**	**FDA approved indications**
**Orlistat** (Xenical®)	USA Approval: 1999	–	120 mg Capsules	$672.99 for 90 capsules	(Xenical®) is a reversible inhibitor of gastrointestinal lipases indicated for obesity management, including weight loss and weight maintenance when used in conjunction with a reduced-calorie diet; also indicated to reduce the risk for weight regain after prior weight loss.
**Benaglutide**	NA	China	1,5,10 mg, SC (other strength available 50, 100 mg)	1 mg EUR 157 5 mg EUR 354 10 mg EUR 571	NA
**Naltrexone-bupropion** (Contrave®)	USA Approval: 2014	–	Extended-Release Tablets: 8 mg naltrexone HCl /90 mg bupropion HCl	Contrave oral tablet, extended-release (90 mg-8 mg) cost between 600 and 700 supplies of 120 tablets	(Contrave®) is a combination of naltrexone, an opioid antagonist, and bupropion, an aminoketone antidepressant, indicated as an adjunct to a reduced-calorie diet and increased physical activity for chronic weight management in adults with an initial (BMI) of 30 kg/m2 or greater (obese) or 27 kg/m2or greater (overweight) in the presence of at least one weight-related comorbidity (e.g., hypertension, type 2 diabetes mellitus, or dyslipidemia).
**Setmelanotide** (IMCIVREE®)	USA Approval: 2020	–	10 mg/mL Injection:	1 mg $119.00 5 mg $356.00 10 mg $607.00 50 mg $1,822.00	(IMCIVREE®) is an (MC4) receptor agonist indicated for chronic weight management in adult and pediatric patients six years of age and older with obesity due to (POMC), (PCSK1), or (LEPR) deficiency confirmed by genetic testing demonstrating variants in POMC PCSK1, or LEPR genes that are interpreted as pathogenic, likely pathogenic, or of (VUS)?
**Tirzepatide** (Mounjaro®)	Expected April 2023	–	injection, solution (prefilled, single-dose pen) 2.5 mg/0.5 mL, 5 mg/0.5 mL, 7.5 mg /0.5mL, 10mg/ 0.5 mL,12.5 mg /0.5mL, 15mg/0.5mL	974 $ 28 days supply for four pens.	Expected April 2023.

**Keys ($):** United States dollar; **(AOM):** anti-obesity medications, **(BMI):** body mass index, **(EUR)**: European commodity, (**GLP-1):** glucagon-like peptide-1, **(LEPR):** leptin receptor, **(MC4):** melanocortin 4, **(NA)**: not available, **(PCSK1):** proprotein convertase subtilisin/kexin type 1, **(POMC):** proopiomelanocortin, **(SC):** subcutaneous, (**USA):** United States of America; **(VUS):** uncertain significance.

**Table 3:** Comparison between Ozempic and Wegovy in specific parameters.

**Parameter**	Ozepmpic® (T2DM)	Wegovy® (AOM)
**Proprietary name**	Semaglutide	Semaglutide
**Pharmaceutical company**	Novo Nordisk	Novo Nordisk
**Posology**	Injection – 2 mg /1.5 mL (1.34 mg/ml)Single-patient-use pens (delivering 0.25 mg or 0.5 mg, or 1 mg per injection). Injection − 4 mg/3 mL (1.34 mg/ml). Single-patient-use pen (delivering 1 mg per injection). Injection − 8 mg/3 mL (2.68 mg/ml).Single-patient-use pen (delivering 2 mg per injection) The dose is increased slowly to 2 mg weekly if required.	o Injection - pre-filled, single-dose pen delivering doses of 0.25, 0.5, 1.7 or 2.4 mg). Dose is increased slowly until a maintenance dose of 2.4 mg weekly is reached.
**Compelling indication**	o As an add-on to diet and exercise to improve glycemic control in adults with T2DM.To reduce the risk of major adverse cardiovascular events in adults with T2DM and established cardiovascular disease.	An add-on to a reduced calorie diet and increased physical activity for chronic weight management in adults with an initial BMI of: - 30 kg/m2 or greater (obesity) or, 27 kg/m2 or greater (overweight) in the presence of at least one weight-related comorbid condition, such as high blood pressure, T2DM, and dyslipidemia.As an adjunct to a reduced calorie diet and increased physical activity for chronic weight management in pediatric patients aged 12 years and older with: - an initial BMI at the 95th percentile or more significant for age and sex (obesity).
**USA approval**	2017	2021

### 3.1 AOM approved for diabetes and/or used off label for weight control

#### 3.1.1 Phentermine

Phentermine, available as [Adipex-P 37.5 mg and Lomaira (8 mg], is among the oldest anti-obesity drug for short-term weight loss. Phentermine can be used for up to 12 weeks in individuals with a BMI of 30 kg/m2 or 27 kg/m2, with at least one medical condition (Tchang et al., 2000). This medication resembles amphetamine, which acts centrally by increasing the release of norepinephrine that suppresses appetite. As a sympathomimetic agent, it increases energy levels, enabling individuals to engage in physical activities. Lean muscle mass loss, metabolic damage, lack of sustainable weight loss, and other side effects have limited the use of this drug. Side effects include headache, dizziness, increased heart rate, palpitations, insomnia, and worsened anxiety. In addition, gastrointestinal side effects such as nausea, constipation, and taste changes may occur. Owing to its sympathomimetic effects, it should be avoided in patients with coronary heart disease and high blood pressure. Before initiating this medication, a cardiac specialist consultation and screening for blood pressure and heart rate were all recommended. In addition, the drug has been avoided in patients with mood disorders, glaucoma, hypothyroidism, and heart attack. Lower doses have been shown to reduce the side effects associated with this drug. The drug has potentially addictive qualities but is less addictive than other amphetamine analogs. The drug should not be consumed with alcohol (Manasrah et al., 2022).

#### 3.1.2 Phentermine/topiramate extended-release (Qsymia)

In 2012, the US FDA authorized using the phentermine/topiramate extended-release combination (Qsymia). The European Medicines Agency (EMA) has declined to approve phentermine/topiramate owing to safety concerns (Sharma et al., 2022). The combination of these two medications has additive effects and reduces the side effects of phentermine. Phentermine is a sympathomimetic that increases the release of norepinephrine (Sharma et al., 2022), while topiramate is a gamma-aminobutyric acid (GABA) modulator. Topiramate also acts as a carbonic anhydrase inhibitor and dopamine/noradrenaline reuptake inhibitor. The exact mechanism of action of topiramate remains unknown. It is recommended for patients with depression. The side effects of this medication include sedation, kidney stones, fatigue, anxiety, lack of focus, and changes in taste (Phentermine, n.d., 2023). A lower dose of 3 mg phentermine plus 23 mg topiramate was better tolerated than higher doses. The most used dose, however, is 7.5 mg phentermine plus 46 mg topiramate. Therefore, the tolerability of this combination should be monitored. In addition, topiramate is associated with fetal cleft palate (a birth defect caused by congenital anomalies). Therefore, it carries a black-box warning that should not be used in pregnant women. Hence, the drug is used cautiously in women of reproductive age (Dhillon, 2022).

The efficacy of this drug was investigated in CONQUER study. The study comprised 2,487 patients with a BMI of 27–45 kg/m2 and two or more cardio-metabolic disorders. Patients were randomly assigned to one of three groups: placebo, low-dose controlled release (phentermine/topiramate CR 7.5/46.0 mg day), or high-dose (phentermine/topiramate CR 15.0/92.0 mg day). The higher dose group, lower dose group, and placebo resulted in weight loss was −10.2 kg, −8.1 kg, and-1.4 kg, respectively. At week 56, the proportion of patients who dropped 5% of their body weight in the treatment group was higher than in the placebo group (21% vs. 62% vs. 70%, respectively). Patients who reduced their body weight by less than 10% had similar results (7% vs. 37% vs. 48%) (Gadde et al., 2011). The CONQUER trial has limited generalizability to all obese patients because the trial included obese patient hat ts with at least two comorbidities. In addition, the trial drug short follow-up duration determines the evidence of the drug's long-term safety. The trial also did not investigate the impact of Qsymia on cardiovascular morbidity and mortality. According to the US FDA, if a weight loss of less than 3% is achieved after three months of treatment, it is recommended to stop the drug or increase the dose. (Gadde et al., 2011).

#### 3.1.3 aLLi (Xenical and Orlistat)

Orlistat (Xenical) was approved in 1999 for weight loss at a dose of 120 mg. It is indicated for use among teenagers and adults (who are overweight or obese. In contrast, aLLii (60 mg pill), a medication with a lower dose for weight loss in overweight adult patients, was introduced to the market in 2007 as an over the counter (OTC) product. The abovementioned medications were used together with a healthy diet and regular exercise. Both reduce fat absorption by inhibiting gastrointestinal and pancreatic lipases, which break down the fat (Cordes et al., 2020). The medication also reduces glucose levels and improves diabetes control. The drug has no impact on the existing adiposity of the body and has no benefit in patients who do not consume fat. This drug is preferred for patients with coronary artery disease or CVDs. Common adverse effects of these medications include hard-to-control stools, gas, oily stools, and loss of vitamins and minerals. Owing to these adverse effects, multivitamin supplementation is required. Patients with trouble absorbing food, pregnant and breastfeeding women, patients who have undergone organ transplants, and patients on cyclosporine should avoid taking these medications. According to the XENDOS study, orlistat lowered the average weight reduction by 4.4 kg from baseline after one year compared to placebo. More than half of orlistat patients (52.8%) and 37.3% of placebo patients lost at least 5% of their body weight (Torgerson et al., 2004). Short follow-up duration, including only obese patients with glucose intolerance, and lack of CV outcome assessment limits this trial's generalizability and outcome interpretation.

#### 3.1.4 Naltrexone sustained release (SR)/bupropion SR (Contrave)

This combination of medications was approved for use in 2014. The neurochemical mechanism by which the naltrexone/bupropion combination causes weight loss remains unknown. The combination is thought to function synergistically in the hypothalamus and mesolimbic dopamine circuits to promote satiety, reduce food intake, and increase energy expenditure. In contrast, bupropion is a dopamine/noradrenaline reuptake inhibitor (Matyjaszek-Matuszek et al., 2018). Naltrexone can manage opioid and alcohol addiction, while bupropion aids in smoking cessation. This drug is associated with nausea, headache, anxiety, and activation. It is recommended for patients with depression. There is a need to titrate the dose gradually to minimize side effects. The use of this drug is not advised in patients with uncontrolled blood pressure, patients diagnosed with bulimia nervosa, seizures, or alcohol withdrawal (Dahlberg et al., 2022). Those with an obesity-related BMI of 30–45 kg/m2 and with cardiovascular risk factors were followed up for 56 weeks as part of the COR-I research. The naltrexone ER and bupropion ER were given in a 32/360 or 16/360 mg ratio. The higher dose and lower dose groups resulted in a drop of 6.1% and 5.0%, respectively; the placebo group, on the other hand, only reduced the body weight by 1.3% (Kühnen et al., 2022). Similar to previous anti-obesity trials, the COR-I trial included a subgroup of the obese population with CVD risk factors and did not evaluate the effect of this drug on the mortality and quality of life of obese individuals.

#### 3.1.5 Liraglutide 3.0 mg (Saxenda)

Liraglutide, a GLP-1 receptor agonist (GLP 1 RA), reduces weight by suppressing appetite and craving, and improving satiety. In 2010, the lower dose of liraglutide, Victoza (liraglutide 1.2 or 1.8 mg/d doses), was approved for managing T2DM.. After four years, Saxenda (liraglutide dose of 3 mg/day) was approved for adults with a BMI of 30 or higher or with a BMI of 27 or higher, and at least one weight-related comorbidity. The age range for Saxenda's prescription was expanded to include obese adolescents aged 12 to 17 years who weighed more than 60 kg in December 2020. A study showed that when combined with moderate-to-vigorous-intensity exercise programs, liraglutide (Saxenda) yielded significantly longer-term weight loss in adults with obesity than either treatment alone at one year. Adverse drug events, gastrointestinal side effects, pancreatic cancer, rarely thyroid cancer, and the need for subcutaneous injection limit the routine use of the drug (Lundgren et al., 2021). The diversity of patients included in the trials of liraglutide, frequent GI adverse effects, and short follow-up trial are some limitations of liraglutide trials for obesity.

#### 3.1.6 Semaglutide (Wegovy®)

Semaglutide 2.4 mg once weekly (Wegovy®) and approved for the long-term treatment of obesity. It is a synthetic form of GLP 1 agonist developed by Novo Nordisk. In 2017, a low-dose variant of this drug (1 mg) was approved for the treatment of T2DM. Semaglutide is a GLP-1 hormone that increases insulin secretion, improves satiety, and slows gastric emptying (Blundell et al., 2017). Wegovy (semaglutide 2.4 mg) was approved by the US FDA in 2021 for the management of excess weight in patients with a BMI of 30 or higher and patients with a BMI of 27 or greater with one or more comorbidities. Wegovy approved based on 10–20% weight reduction depicted in the Semaglutide Treatment Effect in People with Obesity (STEP) clinical program. This drug is chemically modified to stay longer in the body to make individuals feel full for a longer duration than other GLP-1 agonists do. It works similarly to its lower-dose version, ozempic (semaglutide 0.5/1.0 mg), used for diabetic management and sometimes used off-label as a weight loss drug.

The trial (NCT03693430) (Nordisk A/S, 2023a) assessed whether the weight loss effect of once-weekly injections of semaglutide was sustained over a more extended period of 2 years compared to placebo in 304 non-diabetic patients who were obese or overweight. The results showed that semaglutide, in combination with diet and exercise, resulted in a 12.5% greater sustained weight loss from baseline over two years than the placebo. The trial NCT04251156 (Nordisk A/S, 2023b), evaluated the impact of a once-weekly injection of semaglutide 2.4 mg compared to a placebo in 375 overweight or obese participants with or without T2DM from China, the Republic of Korea, and Brazil. This study examined the shift in body weight between the start and end of the study. This study aimed to assess the effects of semaglutide on body weight in people taking it with people taking a “dummy” medicine. In addition to semaglutide, the participants will receive counseling on good eating choices, how to be more physically active, and what else they can do to reduce weight, in addition to taking the drug. The SELECT (NCT03574597) (Nordisk A/S, 2023c) trial is ongoing and aims to evaluate the impact of semaglutide on CVD morbidity and mortality is currently underway. This trial aimed to compare semaglutide 2.4 mg and placebo in 17,500 overweight or obese people without diabetes but with established CVD over 31 to 59 months (Ryan et al., 2020).

Semaglutide 2.4 mg injection is to reduce weight in obese and overweight patients who have a weight-related condition. Wegovy is increasingly used outside of its approved uses, mainly to help people slim down or lose weight. This has led to considerable debate. There are trending posts on social media by individuals who have taken Wegovy with enormous traction because of the claims of weight loss success with negligible risk of drug-related adverse effects such as nausea and headache. However, the manufacturer stated that it does not support or encourage the off-label use of Wegovy for weight loss in patients who were not offered medical advice. The US FDA changed the label of medications used to reduce weight from “weight loss medications” to “anti-obesity medications” to discourage the off-label use of these medications (Gonsahn-Bollie, 2022). Semaglutide has been studied in a series of STEP trials that are conducted in specific locations in the world, which may limit generalizability. In addition, long term trials and evaluating the impact of wegovy on quality of life and patient outcomes is needed,

#### 3.1.7 Tirzepatide (Mounjaro®)

According to phase 2 studies funded by the manufacturer, tirzepatide showed dose-dependent effects on glucose levels and body weight, according to phase 2 studies funded by the manufacturer (Eli Lilly). At the highest doses, it outperformed dulaglutide 1.5 mg/day, albeit in a limited number of participants. The medication was evaluated among diabetes patients in phase 3 trials, either alone or, in conjunction with other treatments. Tirzepatide was administered as a weekly subcutaneous injection at doses of 5 mg, 10 mg, and 15 mg. The SURPASS-1 (NCT03954834) (Rosenstock et al., 2021) trial examined T2DM patients with elevated glycated hemoglobin (HbA1c) levels following dietary and exercise treatments. Weight loss of 6.3 to 8.8 kg was noted after adjusting for a placebo (Rosenstock et al., 2021). The SURPASS-4 (Eli Lilly and Company, 2022a) (NCT03730662) trial compared the safety and efficacy of three tirzepatide dosages (5 mg, 10 mg, and 15 mg) with titrated insulin glargine (metformin, sulfonylurea, or SGLT-2 inhibitor) in more than 2,000 adults with T2DM who have an elevated CVD risk. Most (87%) participants had attended previous events. Compared to insulin glargine, all three doses of tirzepatide significantly reduced HbA1c levels. Additionally, using tirzepatide led to noticeably higher weight reduction and less hypoglycemia (Del Prato et al., 2021). In the SURPASS-5 study (Dahl et al., 2022) (NCT04039503), tirzepatide (5, 10, and 15 mg) was compared with a placebo in T2DM patients receiving insulin glargine with or without metformin. At 40 weeks, glargine plus the highest dose of tirzepatide caused an average HbA1c decline of 2.59%, significantly more significantly than the 0.93% reduction observed with glargine alone. In addition, the tirzepatide group lost an average of 10.9 kg and reduced their insulin dosage, while the placebo group gained an average of 1.7 kg (Dahl et al., 2022). The SURPASS trials are subject to bias because the trials are funded by the manufacturer. Tirzepatide was approved by the FDA in 2022. However, in October 2022, a fast-track designation was processed for an extended indication for the treatment of adults with obesity or overweight with weight-related comorbidities. The results from phase 3 clinical trials (SURMOUNT-1, SURMOUNT-3, and SURMOUNT-4) are expected to be completed during 2023. Recently, the SURMOUNT-2 phase 3, double-blind, randomized, placebo-controlled trial has reported favorable results in weight reduction (Garvey WT et al., 2023). Mounjaro was approved for diabetes but used off-label for weight control.

### 3.2 Emerging AOM for the management of obesity and overweight

#### 3.2.1 Benaglutide

benaglutide, is a recombinant human GLP1 with 100% similarity to human GLP 1(7 3 6) (Zhang et al., 2019). The Chinese FDA approved this drug for treating type 2 diabetes in December 2016. The Chinese guidelines for the prevention and treatment of T2DM recommend beinaglutide as a GLP1RA for the treatment of T2DM (Zhu, 2021). They have been tested in China (Wang et al., 2021, Xin-guo et al., 2011). Beinaglutide, like other GLP1RAs, was efficacious in decreasing HbA1c, fasting plasma glucose (FPG), and postprandial plasma glucose (PPG) in T2DM patients (Wang et al., 2021, Xin-guo et al., 2011). Furthermore, beinaglutide effectively lowered the body weight and BMI of overweight or obese diabetes patients (Wang et al., 2021). A Chinese multicenter, observational, retrospective, open‐label study in 314 subjects with T2DM (between 2017 and 2018) examined the effectiveness of beinaglutide on body weight, HbA1c, blood pressure, and lipid profiles in patients with T2DM. After three months, Beinaglutide significantly reduced body weight (Zhang et al., 2019). Beinaglutide was administered three times a day by subcutaneous injection at different strengths (based on RCT), such as 0.1, 0.2, 0.3, 0.4, 0.5, and 0.6 mg.

#### 3.2.2 Setmelanotide (IMCIVREE®)

Proopiomelanocortin (POMC) and leptin receptor (LEPR) deficiencies are rare hereditary obesity illnesses caused by genetic flaws that interfere with the normal activation of the melanocortin-4 receptor (MC4R) pathway, which governs hunger and satiety signals. It is an MC4 receptor agonist developed for the treatment of obesity arising from POMC, proprotein convertase subtilisin/kexin type 1 (PCSK1), or LEPR deficiency. The MC4R activator setmelanotide (IMCIVREE) was developed specifically for patients with genetic defects that impair MC4R pathway function. Two multicenter, open-label, phase 3 trials are evaluating the safety and efficacy of setmelanotide [(Rhythm Pharmaceuticals Inc., 2021a) NCT02896192 and (Rhythm Pharmaceuticals Inc., 2021b) NCT03287960]. The trial was set to determine the proportion of patients who lost at least 10% of their body weight after one year of treatment with setmelanotide. Important secondary objectives in both studies were the average percentage reduction in weight and appetite from baseline to the end of the trial. Both trials involved a 12-week treatment term with setmelanotide, followed by an eight-week withdrawal period during which patients moved from setmelanotide to a placebo without their awareness. They switched back to setmelanotide after a withdrawal period of another 32 weeks. Participants who completed the entire treatment term were eligible to participate in both trial extension studies. Data from both trials revealed that after one year of treatment, 80% of patients with POMC deficits and 45.5% of those with LEPR deficiency lost more than 10% of their body weight. The most frequent side effects in the POMC experiment were injection site response and hyperpigmentation, which all ten participants reported. Five and three participants recorded nausea and vomiting, respectively. All 11 participants in the LEPR experiment reported injection site responses, skin disorders by five participants, and nausea by four as the most common treatment-related side effects. In both trials, no significant treatment-related side effects were observed (Clément et al., 2020). Setmelanotide was licensed by the US Food and Drug Administration in 2020 for chronic weight management in adult and pediatric patients aged ≥ 6 years with POMC, LEPR, or PCSK1 deficiency. After the phase, three studies showed that Setmelanotide treatment resulted in an average BMI loss of 7.9% for the 44 BBS participants, which was extended in 2022 to include people with Bardet-Biedel syndrome (BBS).

#### 3.2.3 Tesofensine

The clinical trial (NeuroSearch A/S, 2013) NCT00394667 evaluated tesofensine, a drug that blocks the presynaptic neurotransmitters uptake such as noradrenaline, dopamine, and serotonin. Tesofensine 0.5 mg is shown to be a more potent weight loss medication than other drugs. However, the findings need to be ascertained in phase 3 trials (Astrup et al., 2008). Both doses of Tesofensine did not significantly increase blood pressure than the placebo (Astrup et al., 2008).

#### 3.2.4 Fibroblast growth factor-21 (FGF-21)

High levels of FGF21 in the blood are characteristic of obesity in rats and humans. Resistance to FGF21 is hypothesized to be a component of obesity-related endocrine changes. The resistance in obese mice and patients' adipose tissue is due to abnormally low levels of the FGF co-receptor Klotho, which is necessary for FGF21 cellular action. However, when utilized as a pharmaceutical product to treat obese rodents and humans, native FGF21 and FGF21 derivatives preserve their, sound metabolic and weight-loss effects. FGF21 derivatives or compounds that imitate FGF21 function appear to be promising candidates for developing new AOM that boost energy expenditure. Phase one trial funded by Novo Nordisk (Novo Nordisk A/S, 2019) (NCT03479892) is underway for weight control in overweight or obese people (Novo Nordisk A/S, 2019).

#### 3.2.5 Beta 3 adrenergic agonist (3 AR)

Kern's 2020 (NCT02919176) study showed improved glucose control in mice’s beige adipose tissue. It has been anticipated that 3-AR agonist, mirabegron treatment of obese, insulin-resistant individuals, can enhance beige adipose development in subcutaneous white adipose tissue (SC WAT). Mirabegron therapy significantly enhances glucose homeostasis Inhibiting SC WAT with mirabegron improves muscle oxidative capacity and cell function (Finlin et al., 2020).

#### 3.2.6 GLP1R/GCGR/GIPR triple agonist

A phase 1 trial (Novo Nordisk A/S, 2018) (NCT03095807) is being conducted to determine the safety and tolerability of single subcutaneous doses of NNC9204-1706 in overweight or obese male patients. However, results still needed to be obtained. The drug combines the effects of GLP-1, glucagon (GCG), oxyntomodulin, glucose-dependent insulinotropic peptide (GIP), and peptide YY to reduce weight further. Drugs that agonize GLP1R/GCGR and GLP1R/GIPR are under development. The triple agonism of GLP1R/GCGR/GIPR is currently under investigation (Novo Nordisk A/S, 2018).

#### 3.2.7 Dual GLP1R/GCGR/GIPR agonist

The NCT03308721 trial (Novo Nordisk A/S, 2018) examines a new study drug for weight loss in overweight or obese patients. The goal was to explore the impact of the drug and how the studied drug interacts with the body, and how it is eliminated. NNC9204-1177 (Novo Nordisk A/S, 2021) (the new research drug) or placebo will be administered to participants (a formula that looks like the study medicine but does not have active ingredients) (Novo Nordisk A/S, 2021).

#### 3.2.8 Retatrutide (Triple mechanism, GLP1/GIP/GR)

A new emerging molecular entity, retatrutide (LY-3437943), is being developed for diabetes patients with obesity. It was administered once daily subcutaneously. This drug candidate targets glucagon-like peptide 1 (GLP-1), glucose-dependent insulinotropic polypeptide (GIP), and glucagon receptor (GR). The FDA set this year for approval. The drug is dispensed only through prescription. Although it shares some similarities with other AOMS, such as tirzepatide and semaglutide, it was claimed to have a greater effect on weight reduction. However, the price of a drug is too high (Coskun et al., 2022).

Retatrutide was evaluated in phase 2, a double-blind, placebo-controlled RCT involving 338 adults with a BMI of 30 or higher or a BMI of 27 to less than 30 plus at least one weight-related condition (Jastreboff et al., 2023). Subjects received subcutaneous retatrutide (1 mg, 4 mg [initial dose, 2 mg], 4 mg [initial dose, 4 mg], 8 mg [initial dose, 2 mg], 8 mg [initial dose, 4 mg], or 12 mg [initial dose, 2 mg]) or placebo once weekly for 48 weeks. The primary endpoint was the percentage change in body weight from baseline to 24 weeks. Secondary endpoints included the percentage change in body weight from baseline to 48 weeks and a weight reduction of 5% or more, 10% or more, or 15% or more. Safety was also assessed. The least-squares mean percentage change in body weight at 24 weeks in the retatrutide groups was − 7.2% in the 1-mg group, −12.9% in the combined 4-mg group, −17.3% in the combined 8-mg group, and − 17.5% in the 12-mg group, as compared with − 1.6% in the placebo group. At 48 weeks, the least-squares mean percentage change in the Retatrutide groups was − 8.7% in the 1-mg group, −17.1% in the combined 4-mg group, −22.8% in the combined 8-mg group, and − 24.2% in the 12-mg group, as compared with − 2.1% in the placebo group. Retatrutide was evaluated at 48 weeks, and a weight reduction of 5% or more, 10% or more, and 15% or more had been reported. The weight reduction occurred in 92%, 75%, and 60%, respectively, of the participants who received 4 mg of retatrutide; 100%, 91%, and 75% of those who received 8 mg; 100%, 93%, and 83% of those who received 12 mg; and 27%, 9%, and 2% of those who received placebo. Retatrutide treatment for 48 weeks substantially reduced body weight in adults with obesity, Funded by Eli Lilly; ClinicalTrials.gov number, NCT04881760, (Jastreboff et al., 2023).

#### 3.2.9 Orforglipron

The glucagonlike peptide-1 (GLP-1) receptor agonist (RA), the experimental drug orforglipron (non-peptide) was pivoted by the researcher (Funded by Eli Lilly; GZGI ClinicalTrials.gov number, NCT05051579) as a once-daily oral therapy for weight reduction in adults with obesity. A recent phase 2 double-blind RCT enrolled 272 adults with obesity or overweight plus at least one weight-related coexisting condition without diabetes. Subjects received orforglipron at one of four doses (12, 24, 36, or 45 mg) or placebo once daily for 36 weeks, with the percentage change from baseline in body weight assessed at weeks 26 and 36 as primary and secondary endpoints, respectively. The mean change from baseline in body weight ranged from − 8.6% to − 12.6% in the orforglipron cohort versus − 2.0% in the placebo group and from − 9.4% to − 14.7% with orforglipron versus − 2.3% with placebo, at week 26, At week 36, respectively. The orforglipron was discontinued in 10 to 17% of participants across dose cohorts. The drug improved both the weight and metabolic features of obesity (Wharton S et al. 2023).

### 3.3 Other innovative immerging obesity therapies

New strategies, such as gene and cell therapies, are being considered to treat obesity. Gene therapy aims to increase or decrease gene products in favor of fat breakdown and energy expenditure, resulting in weight loss. Ex vivo gene therapy technique was used to generate and implant cells that express the CPT1AM protein, which made it possible to reduce weight of obese mice. Other genes therapies investigated for obesity include Follistatin (FST) (Jimenez et al. 2018), Fibroblast growth factor 21 (FGF21) (Lapik et al. 2016), Adipose-derived mesenchymal stem cells (ADMSCs) (Lopez et al. 2023), and CRISPR-Cas9-based gene editing system (Jayachandran et al. 2023). Recent research found that personalized recombinant leptin therapy helped correct severe, early-onset obesity linked with leptin genetic variations in two kids (Funcke et al., 2023). There are safety concerns regarding these therapies, and ongoing research on delivery methods for these therapies.

### 3.4 Obesity, shortage of AOM, telehealth, and social media

A study investigating the impact of social media use on weight loss programs showed that incorporating social media as part of interventions to reduce weight can be a cost-effective social support system that increases client engagement (Jane et al., 2018). Social media use could have its advantages and disadvantages in the use of anti-obesity medications. While social media use can increase patient adherence to treatment and improve patient outcomes, it can also lead to misinformation and the inappropriate use of medication. The recent TikTok trend has been blamed for the increased improper use of ozempic for weight loss, which led to a shortage of this antidiabetic drug (Choi and Vu, 2023). Antidiabetic medications that can reduce body weight, such as munjurno (trizeptide) and Qzembic (semaglutide), are available as non-prescription drugs. The social media trend that promotes drug effectiveness to reduce weight has resulted in an excessive supply demand globally, including in the UAE. This trend in using antidiabetic drugs for weight loss has limited access to patients with diabetes who need these medications the most. In addition, the use of drugs that reduce weight requires proper consultation with healthcare providers to avoid potential drug interactions and toxicity and to ensure sustainable weight loss. Further research is needed to understand the influence of social media on weight loss drug use and to determine the best way to use social media to support weight loss interventions (Al Shouk, 2023).

In 2022, a large demand pushed Novo Nordisk's diabetes drug Ozempic and the weight loss drug Wegovy (semaglutide) into shortage. Novo Nordisk replenished supplies of Ozempic, and social media-driven enthusiasm over the drug’s use for weight loss led to six months of shortages. Social media and telehealth have placed an enormous burden on the demand for ozempic, a prescription to treat T2DM. The present medical scarcity has restricted availability for people with T2DM who rely on it to control their blood sugar levels. Digital health facilitated the delivery of prescription ozempic. The market has grown significantly with popular ozempic procurement. The ozempic market outperformed forecast projections for 2022 and is likely to grow to approximately $2 billion by 2023 (Choi and Vu, 2023). The story of Wegovy is not far from this, as the popular weight loss drug Wegovy has been in short supply in pharmacies across the USA (increase in demand combined with manufacturing hiccups) due to the impact of massive global media information (Lovelace Jr., 2023).

Mounjaro’s story is not exceptional, going through the same phases (driven by celebrities and influencers) of massive misuse (dynamic demand), shortage (two-month-long shortage), replenishing (intermittent delays), keeping patients waiting, and ending with a recycling wheel. The USA health regulator has added Mounjaro to its list of drugs facing shortages and highlighted that Lilly's manufacturing firm struggles to meet the booming demand for the newly approved diabetes injection (Reuters, 2023). The prospect of FDA approval of AOM is based on the results of recent trials. Such as SURMOUNT 1–4 (Eli Lilly and Company, 2023a, Eli Lilly and Company, 2023b, Eli Lilly and Company, 2022b, Jastreboff et al., 2022 and STEP 1–8 trials (Davies et al., 2021, Garvey et al., 2022, Kadowaki et al., 2022, Nordisk A/S, 2023b, Rubino et al., 2021, Rubino et al., 2022, Wadden et al., 2021, Wilding et al., 2021). Older trials SCALE, SEQUEL, QUIP, CONQUER, COR-I, and XENDOS trials (Allison et al., 2012, Gadde et al., 2011, Garvey et al., 2012, Greenway et al., 2010, Pi-Sunyer et al., 2015, Torgerson et al., 2004). The results of trials with FDA-approved AOM are shown in [Table 1]. As referenced (Allison et al., 2012, Davies et al., 2021, Eli Lilly and Company, 2022b, Eli Lilly and Company, 2023a, Eli Lilly and Company, 2023b, Gadde et al., 2011, Garvey et al., 2012, Garvey et al., 2022, Greenway et al., 2010, Jastreboff et al., 2022, Kadowaki et al., 2022, Nordisk A/S, 2023b, Pi-Sunyer et al., 2015, Rubino et al., 2021, Rubino et al., 2022, Torgerson et al., 2004, Wadden et al., 2021, Wilding et al., 2021). The pharmacoeconomic comparative cost analysis for FDA-approved AOM was depicted in [Table 2**, internet**].

### 3.5 Monitoring of approved AOM

A multisite study review of patients’ medical records has recommended monitoring AOM for adverse events (Pearl et al., 2023, Valladales-Restrepo et al., 2023, Calderon et al., 2022).

The following monitoring parameters might facilitate the safe utility of AOM.

1. Regular monitoring of heart rate (a symptom of palpitation); discontinue Saxenda® in patients with high persistent heart rate.

2. Mood changes and suicidal thoughts are associated with Saxenda.

3. Monitor blood glucose pre and post-therapy of WEGOVY®.

4. Monitoring of liver function, lipid profiles, and renal function was required for all patients.

5. Renal function must be monitored while starting or increasing Wegovy® doses in patients with severe adverse gastrointestinal responses, and renal impairment.

6. In addition, patient compliance with AOMs and weight loss progress should be monitored.

7. Adherence to AOM dosing and duration is required in compliance with prescriber and prescribing guidelines.

### 3.6 Counseling patients: The role of healthcare professionals (HCPs)

Individuals with AOM should be counseled via discussion forums and conversations regarding weight management and options that fit their daily routines. A study showed that two-thirds of obese patients want their HCPs to initiate a conversation about weight. The correct framework can help guide these discussions with everyone. The HCPs should show empathy, and reflective listening, encourage a 2-way dialogue, use to list, and review current progress, recognize patients’ efforts, identify future steps, and follow up with their patients during weight management sessions. Trained and authorized HCPs can counsel patients on the appropriate indications of AOMs, their risks, benefits, drug interactions, compliance, and the need for lifestyle changes needed to harness the best benefits of AOMs.

### 3.7 Summary of the key findings

Obesity is a complex chronic disease that negative individuals' emotional, physical, and social well-being. Additionally, public health problems are associated with various medical issues. Being overweight or obese is a complex, progressive, and chronic problem. Weight loss interventions for reducing excess weight need to be initiated as early as possible, individualized, and maintained. A combination of lifestyle intervention, pharmacotherapy, and surgery is needed for significant weight loss. Modest weight loss has a significant medical benefit. Before the use of any AOM, there is a need to ensure the drug indicated is likely to be effective, safe, and affordable by the patient. Continuous accountability and follow-up are needed to ensure effective and sustainable weight loss.

Older generation AOM only results in modest weight loss and is associated with several adverse effects. Recently, approved the use of GLP-1 RA (e.g., semaglutide) and off-label use of GLP1-GIP 1 agonists (e.g., tirzepatide) resulted in significant weight loss that is comparable to weight loss with gastric bypass surgery. The drugs act by suppressing appetite and improving satiety and are well tolerated. While semaglutide is approved as an anti-obesity (Wegovy) and antidiabetic (ozempic) drug at different doses, tirzepatide is currently only approved for diabetes.

The drugs are recommended for weight loss in patients who are obese or who are overweight and have a weight-related medical problem. The major limitations of the routine and sustained use of AOM are its excessive cost, safety concerns, knowledge of indications, healthcare provider training, stigma, and insurance coverage. However, the significant weight loss linked to these drugs has increased demand that exceeds the supply in some countries. This trend raises a concern about diverting the use of these drugs from patients who need them to treat diabetes or obesity.

A growing concern about AOM is its cardiovascular safety. However, only Ozempic (semaglutide antidiabetic form) is approved to lower the risk of a major cardiovascular event (like a heart attack or stroke) in patients with type 2 diabetes. Nevertheless, although studies are ongoing, Mounjaro (tirzepatide) has yet to receive this indication. The long-term safety of AOM deserves further research and evaluation with post-marketing surveillance studies.

### 3.8 Status and future of obesity management

Currently, obesity is identified as a chronic disease, and there are several AOMs used to manage obesity. Collaborative effort is required to manage this disease optimally. Enhancing individualized therapy that considers individual needs and preferences is necessary. The effectiveness of available AOMs is variable and permanent solutions are not available. Various issues, such as high cost, limited availability of the AOMs, and lack of insurance coverage need to be addressed before routine use of these drugs. Further research is ongoing to create safer, more effective AOMs. Combination of medications, lifestyle modifications, and behavioral changes could be the way forward in the management of obesity. In addition, more data on long-term efficacy and safety is needed on the effect of newly approved AOMs. In addition, there is a need for enhancing research on the promising targets of gene and cell therapies. Social media platforms and telemedicine can be used to disseminate the right patient education on obesity.

### 3.9 Strength and weakness

The strength of our narrative review is that it is more comprehensive than a systematic review, as it is not limited to a predetermined set of inclusion criteria. Therefore, the current narrative review may serve as a useful tool for exploring emerging or complex areas of chronicity, such as obesity and overweight, that are extensively studied. However, potential biases exist, for instance, in the selection and interpretation of the literature, as it is at the author's discretion. We acknowledge no potential limitations or conflicts of interest in the current narrative review.

## 4 Conclusion

In recent years, the use of AOM has increased enormously despite its sometimes-dubious safety and ineffectiveness. We have highlighted the currently proven AOM; off-label ones, recent advances, and future medications, and presented the potential challenges ahead. The use of AOM is challenged by concerns about safety, sustainable effects, access, misuse, and excessive cost. AOM use needs to be individualized and use along with lifestyle modifications. Long-term AOM medications are taken after proper consultations and with ongoing monitoring. The public and medical inertia should be vigilant to the real-world benefits of anti-obesity drugs and their achieved desired effectiveness with an improved safety profile.

## 5 Authors' contributions

We declare that all of the authors have made substantial contributions to the conception, and design of the work; the acquisition, analysis, and interpretation of data, drafted the work, and revise it critically for important intellectual content. In addition to approval of the version to be published; and agreed to be accountable for all aspects of the work in ensuring that questions related to the accuracy or integrity of any part of the work are appropriately investigated and resolved.

## Declaration of Competing Interest

The authors declare that they have no known competing financial interests or personal relationships that could have appeared to influence the work reported in this paper.
